# Chemotaxis of Cell Populations through Confined Spaces at Single-Cell Resolution

**DOI:** 10.1371/journal.pone.0029211

**Published:** 2012-01-18

**Authors:** ZiQiu Tong, Eric M. Balzer, Matthew R. Dallas, Wei-Chien Hung, Kathleen J. Stebe, Konstantinos Konstantopoulos

**Affiliations:** 1 Department of Chemical and Biomolecular Engineering, Johns Hopkins University, Baltimore, Maryland, United States of America; 2 Johns Hopkins Institute for NanoBioTechnology, Johns Hopkins University, Baltimore, Maryland, United States of America; 3 Johns Hopkins Physical Sciences-Oncology Center, Johns Hopkins University, Baltimore, Maryland, United States of America; 4 Center of Cancer Nanotechnology Excellence, Johns Hopkins University, Baltimore, Maryland, United States of America; 5 Department of Chemical and Biomolecular Engineering, University of Pennsylvania, Philadelphia, Pennsylvania, United States of America; University of Illinois at Urbana-Champaign, United States of America

## Abstract

Cell migration is crucial for both physiological and pathological processes. Current *in vitro* cell motility assays suffer from various drawbacks, including insufficient temporal and/or optical resolution, or the failure to include a controlled chemotactic stimulus. Here, we address these limitations with a migration chamber that utilizes a self-sustaining chemotactic gradient to induce locomotion through confined environments that emulate physiological settings. Dynamic real-time analysis of both population-scale and single-cell movement are achieved at high resolution. Interior surfaces can be functionalized through adsorption of extracellular matrix components, and pharmacological agents can be administered to cells directly, or indirectly through the chemotactic reservoir. Direct comparison of multiple cell types can be achieved in a single enclosed system to compare inherent migratory potentials. Our novel microfluidic design is therefore a powerful tool for the study of cellular chemotaxis, and is suitable for a wide range of biological and biomedical applications.

## Introduction

Cell migration plays an important role in diverse (patho)physiological processes, including inflammation, wound healing, angiogenesis and cancer metastasis [1], [2], [3], [4]. Accordingly, cell migration is studied in an experimental setting to better understand the biological mechanisms of locomotion in human health and disease. *In vivo* assays of cell migration require the use of sophisticated microscopic techniques on live animals that are technically challenging and expensive [5], [6]. On the other hand, commonly used *in vitro* assays, such as the modified Boyden chamber or transwell assay provide end-point data but no information on cell behavior between the start and conclusion of the experiment [7], [8]. The wound-healing assay is another popular *in vitro* method for measuring cell motility on planar two-dimensional (2D) surfaces [8]. Although this assay provides real-time data, it requires a confluent cell monolayer, which precludes the analysis of individual cell movement, and is incompatible with cell types that do not naturally form monolayers. Furthermore, wound-healing experiments suffer from temporal limitations, are complicated by the effect of cell doubling, and fail to incorporate a chemotactic stimulus for the study of directed cell locomotion.

Extracellular matrix (ECM) substrates influence cell differentiation and function through both biochemical content and physical configuration [9]. To replicate the influence of *in vivo* microenvironments *in vitro*, reconstituted ECM gels have been utilized to study cell motility in three-dimensional (3D) settings [10], [11]. However, this technique suffers from multiple drawbacks, including poor antibody delivery to fully immersed cells, nonspecific interactions between applied reagents and ECM components, and suboptimal properties for microscopic analysis. Additionally, there is inherent variability in the structure and density of ECM fibers between preparations, so embedding cells into matrix in a repeatable fashion can be technically challenging. Moreover, the lack of well-defined chemotactic gradients in these systems means current ECM gel studies fail to induce cell migration along a pre-specified trajectory; instead these assays observe random cell movements.

Molecular gradients are an inherent feature of mammalian physiology that are necessary for the activation of intracellular signaling events and establishing directional cues for migrating cells. Microfluidic technique has been utilized to generate gradients for *in vitro* cell migration studies [12], [13], [14]. Specifically, microfluidic technology has enabled researchers to apply controlled chemotactic gradients to migratory cell populations on 2D substrates [15], [16], [17], [18], [19], [20], [21], [22]. However, it has recently been acknowledged that flat substrates are of limited physiological relevance. Thus, many investigators have sought ways to increase the dimensional complexity of *in vitro* migratory environments. To address this issue, polydimethylsiloxane (PDMS)-based microfluidic channels have been developed for the study of cellular movement in confined spaces. This technique has been used to examine the locomotion of leukocytes and tumor cells [23], [24], [25], [26], [27], [28]. However, these devices do not incorporate a controlled chemotactic stimulus, and therefore are useful only for the study of spontaneous migration [24], [27], [28]. As no controlled chemotactic gradient can be administered in 3D gel assays, the solution to this problem has thus far required the use of external pumps and cumbersome tubing setups [25], [26].

In this work, we offer a key improvement of the PDMS microchannel device by incorporating an inclusive method for establishing a diffusion-driven chemotactic gradient. The gradient is established by positive pressure between inlet and outlet wells, and does not require the use of external pumps. By exploiting the principles of microfluidic dynamics and capillary forces [29], [30], laminar flow enables effective diffusion and ensures a steep gradient is maintained for at least 9 h. In addition, unlike traditional Boyden chamber assays that allow only a single pore dimension to be evaluated, we fabricated the migration chamber with multiple channel sizes ranging from 3×10 µm to 50×10 µm (width×height). Furthermore, this device allows the user to directly compare phenotypes of cell migration in a variety of physical geometries within a single device; these include narrow channels (≤6 µm in width) that approximate physiological environments. Our PDMS migration chamber also enables the user to track the migration of whole cell populations and individual cells in real-time at maximum optical resolution, making it amenable to static and real-time high-resolution microscopic analyses. The user can modify microchannel size, ECM type, or the inclusion of small molecules and pharmacological inhibitors to study their effect on cell migration. Finally, we analyze the migration of metastatic and non-tumorigenic breast epithelial cells, and demonstrate our ability to distinguish them based on their distinct inherent migratory potential. This demonstrates that the device can be used to directly compare different cell types, minimizing experiment-to-experiment variation and enabling the user to study how mixing hetergenous cell populations influences their migration through paracrine interactions.

## Results

### Fabrication and Characterization of the Microfluidic Migration Chamber

**Figure 1:**
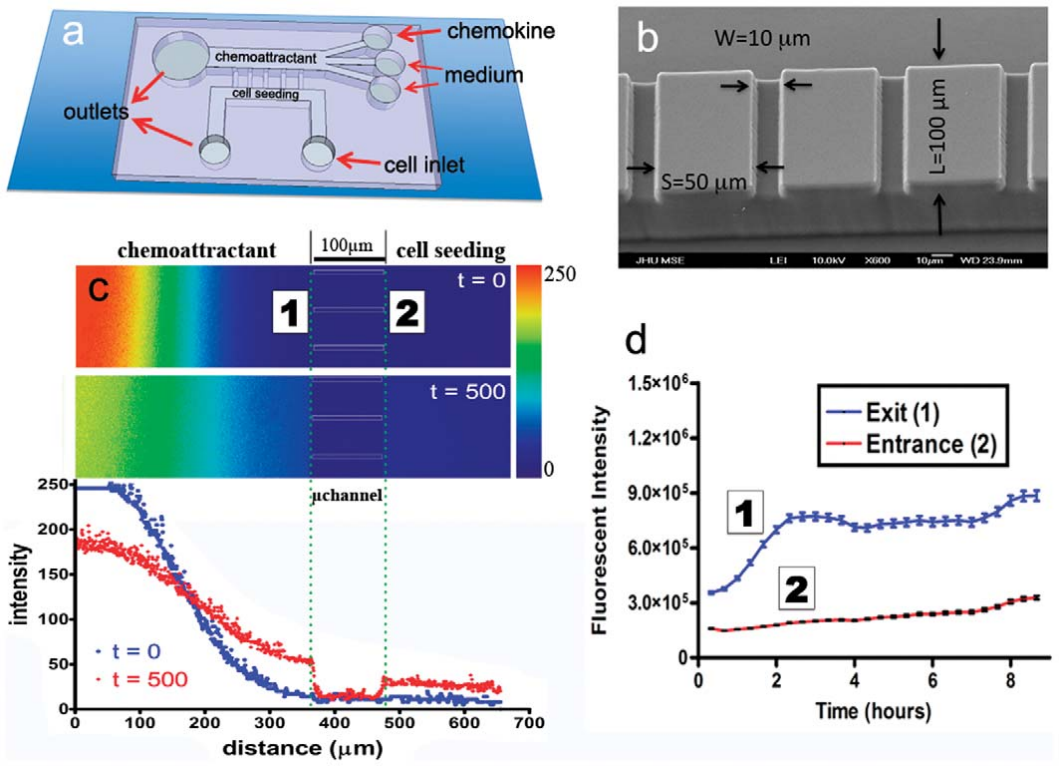
Design and characterization of the microfludic cell migration chamber. a) Schematic of cell migration chamber. The microfluidic device consists of an array of microchannels of varying width, ranging from 3 µm to 50 µm. b) A representative SEM image of vertical microchannels spaced 50 µm apart. c) Quantitative heatmap of fluorescence intensity indicates the stability of a chemokine-like gradient. The lower panel is a quantitative representation of fluorescence signal decay plotted as arbitrary intensity units as a function of distance from chemokine stream over the course of 9 h. The microchannel position is indicated by dotted green lines, which corresponds to a drop in fluorescence intensity due to occlusions of the FITC-dextran by the PDMS walls. d) Fluorescence intensity measurement over time at either sides of the microchannels, i.e., cell entrance (2) and cell exit (1) regions.

The microfluidic migration chamber was fabricated by standard lithographic techniques (Figure S1) [31], [32]. In brief, a photoresist master defining the microchannel geometry was produced by photolithography. PDMS was used to produce a negative mold of this master, and subsequently sealed to a glass slide via brief oxygen plasma treatment [33] to form an enclosed microfluidic device (Figure 1a) of overall dimensions L_D_ = 3.5 cm×W_D_ = 2 cm×H_D_ = 0.5 cm. Parallel microchannels separated by a distance of S_C_ = 50 µm were designed of height H_C_ = 10 µm and length L_C_ = 200 µm with various widths W_C_ ranging from 3 to 50 µm (Figure 1b). The microchannels were aligned in a ladder-like configuration orthogonally to and connected to two larger main channels which serve as a cell seeding source and a chemokine reservoir.

Scanning electron microscopy was used to compare the desired feature dimensions with the actual size of the photoresist master (Figure S2) and PDMS microchannels (Figure 1b). Experimental variation of feature sizes was within 1–2% for channel widths of 6 µm or greater, with less than 15% variability of 3 µm features. The gradient-generating portion of the microfluidic device consists of 3 inlet reservoirs that feed into a large central channel (Figure 1a). Each of these inlets provides a source of laminar flow that is driven by positive pressure differentials between the inlet and outlet wells. The chemoattractant is placed in the uppermost inlet, whereas the lower two receive serum-free medium (Figure 1a). This configuration results in three parallel laminar streams with a maximum concentration of chemoattractant molecules in the topmost stream. Chemoattractant molecules steadily diffuse towards the cell seeding chamber (Figure 1a), thereby rapidly establishing a gradient across the microchannels (Figures 1c and 1d). To visualize the diffusion process, fluorescent FITC-conjugated dextran was introduced at the uppermost inlet reservoir as a chemokine mimetic [21]. The gradient is rapidly established and maintained for at least 9 h (Figures 1c and 1d). To demonstrate the general utility of our migration chamber for cell motility studies, we performed experiments with multiple cell lines, including human osteosarcoma cells (HOS), human breast adenocarcinoma cells (non-metastatic MCF-7 and metastatic MDA-MB-231) and non-tumorigenic mammary epithelial cells (MCF-10A).

### Dynamic Optical Range for Simultaneous ‘Population’ and ‘Single-Cell’ Study

**Figure 2:**
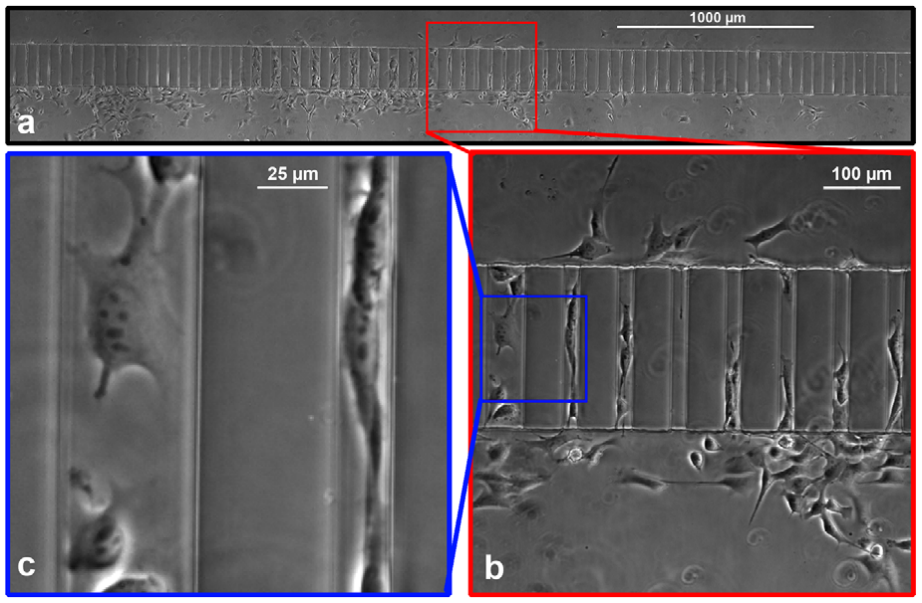
Dynamic optical range for variable-scale analysis of cellular behavior. a) A panoramic micrograph was automatically stitched from multiple adjacent fields of view to visualize the microchannel array. (Pictured: HOS cells after 5 h of migration). b) A single field of view from the stitched montage shown in panel (a), captured with a 10× objective. (Pictured: cells migrating in 50 µm and 20 µm microchannels). c) A cropped section of the image in panel (b) shown at 100% zoom.

With the use of a motorized microscope stage, multiple fields of view could be stitched together in real-time to provide data on hundreds of cells simultaneously (Figure 2a). Individual fields of view can be selected for analysis to provide a more detailed examination of cell behavior in specific microchannel configurations (Figure 2b). Displaying a section of this field of view at 100% zoom indicates that individual cells can be readily visualized (Figure 2c). In this manner, a single dataset can provide real-time information on population-scale cell movement (Movie S1) and individual cell migration (Movie S2). Therefore, our technique is a versatile tool suitable for a wide range of research applications.

### Microchannel Dimensions Affect Cell Morphology and Migration Speed

**Figure 3:**
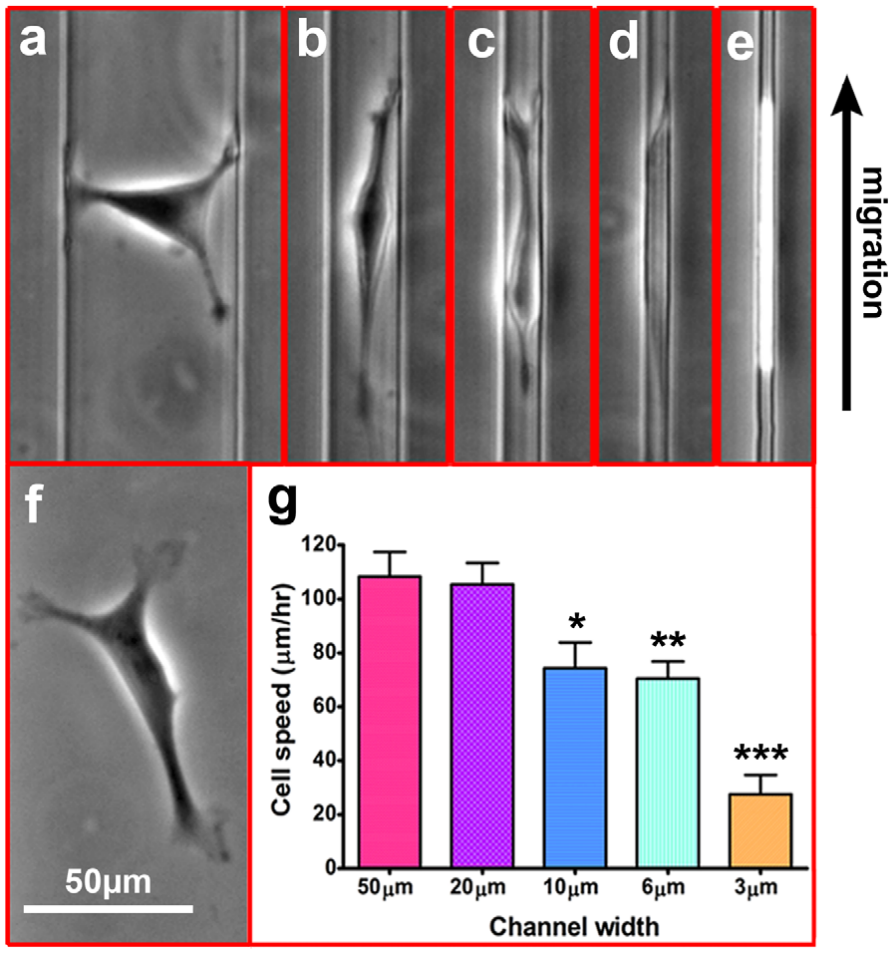
The influence of channel width on migratory cell morphology and migration speed. HOS cells are pictured as they migrate ‘upward’ toward a chemokine source through microchannels of various widths. Cells migrating in 50 µm (a) and 20 µm (b) -wide channels exhibit morphologies similar to cells on a planar surface (f). Cells migrating through 10 µm (c) and 6 µm (d) channels are laterally restricted by the channel walls. Cells migrating through 3 µm-wide channels (e) undergo significant deformation, becoming uniform and morphologically de-polarized. Scale bar for (a–f) is indicated in panel (f) as 50 µm. g) Cell migration speed inside different channels of each dimension was calculated, and shown to be influenced by microchannel width (error bars depict standard error of the mean).

Within a single migration chamber, HOS cells encounter arrays of microchannel of sizes both above (20 and 50 µm) and below (3, 6, and 10 µm) the average cell diameter of 12 µm. This heterogeneous internal configuration enables the researcher to assess the effects of physical confinement on various aspects of cell migration. To demonstrate the effect of microchannel dimensions on cell morphology during chemotaxis, HOS cells were seeded and allowed to migrate through type I collagen-coated microchannels (Figure 3). Cells within 50 µm-wide (Figure 3a) and 20 µm-wide (Figure 3b) microchannels were not constricted by the PDMS walls, and were morphologically comparable to cells on a flat 2D surface (Figure 3f). Cells migrating through microchannels 10 µm or less in width experienced physical confinement by lateral channel walls, and changed their morphology in order to squeeze and move through the channels. Specifically, cells migrating in 10 µm (Figure 3c) and 6 µm (Figure 3d and Movie S2) microchannels deformed inwardly to assume an ellipsoid cell shape, and formed thin protrusions of the leading edge which continually probed the channel surfaces ahead of the advancing cell. Cells within 3 µm-wide microchannels deformed even more dramatically (Figure 3e and Movie S2). Narrow microchannels induced cell contact with all four ECM-coated channel surfaces, and therefore mimic the 3D microenvironment. Interestingly, when cells were migrating through ‘3D-like’ microchannels, discrete leading-edge protrusions typically associated with 2D migration were lost; rather both the leading and trailing edges showed similar morphology and uniformly filled the volume of the channel.

To assess the effect of physical constriction on cell migration, directional cell velocity was quantified for each microchannel width (Figure 3g). Inside 50 and 20 µm-wide microchannels, no significant differences in average HOS cell migration speeds were observed (108±9 and 105±8 µm/hr, respectively), which correlate with the similar cell morphologies observed for these channels. Cells migrating through 10 and 6 µm-wide microchannels displayed similar cell migration velocities (74±9 and 70±6 µm/hr, respectively), but were significantly reduced relative to those in 50 µm-wide channels. Moreover, cells migrating through 3 µm-wide channels retained an average 70% reduction in migration speed relative to 50 µm-wide channels. Interestingly, HOS cells largely exhibited persistent unidirectional migration in all channel widths, independent of channel length which was varied from 100 to 400 µm (Figure S3a), owing to the stability of the chemotactic gradient demonstrated in Figure 1. To ensure hypoxia, especially inside 3 µm wide microchannels, does not influence cell migration ability, we performed a mathematical calculation of oxygen depletion rate inside the microchannel. We found that gas transfer rate to microchannels is sufficient to compensate for the cell-mediated oxygen consumption rate due to gas permeability nature of PDMS.

**Figure 4:**
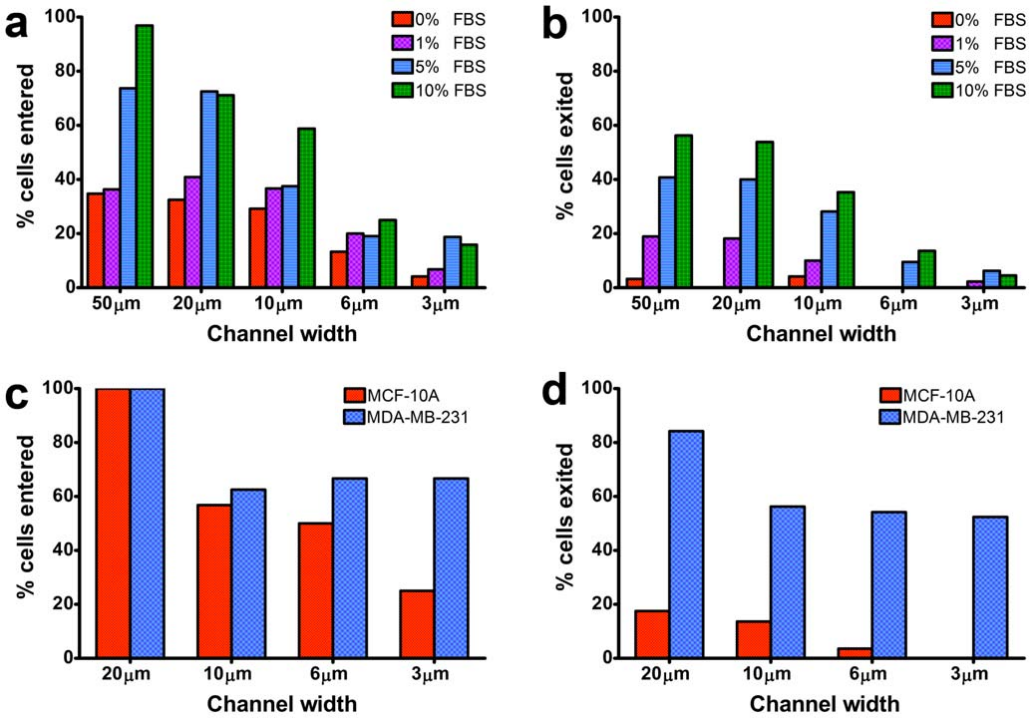
Effect of chemokine concentration on cell entrance and exit from microchannels. A range of FBS concentrations (0%, 1%, 5% and 10%) were tested for the ability to induce cells to enter and migrate successfully through the full channel length (200 µm), and exit the microchannels. The rate of HOS cells that migrated into (a) and exited from (b) microchannels is expressed as percentage of the total number of cells seeded within a distance of 100 µm from the channel entrances. 10% FBS was used to examine the migration rate of MDA-MB-231 and MCF-10A that entered (c) and exited (d) the 200 µm long microchannels in order to compare their respective invasive potential.

To further examine the functional consequence of the chemoattractant gradient, we inspected cell migration speed in response to a range of FBS concentrations by comparing the frequency with which cells successfully entered and exited each microchannel. FBS contains a number of chemoattractant molecules which makes it suitable for chemotaxis studies with a wide range of cell lines [34]. At an FBS concentration of 10% over the course of the 10 h experiment, we observed that 96% of HOS cells seeded within 100 µm of the microchannel entrances entered the 50 µm-wide channels (Figure 4a), while a fraction of them (56%) successfully exited the opposite end (Figure 4b). A reduced fraction of cells (58%) were able to enter the 10 µm-wide microchannels, while only 35% of those seeded cells exited these channels. This trend continued for 6 µm and 3 µm-wide microchannels, and was dependent on the concentration of FBS applied to the gradient for all microchannel sizes (Figures 4a and 4b). Experiments with additional cell lines indicated that both metastatic MDA-MB-231 breast carcinoma cells and non-tumorigenic MCF-10A mammary epithelial cells were able to penetrate microchannels of 6 µm or greater in width with similar efficiency (Figure 4c), but only the metastatic MDA-MB-231 cells efficiently exited the microchannels (Figure 4d). Of note, only the metastatic MDA-MB-231 cells were capable of squeezing and moving completely through the 3 µm-wide microchannels (Figures 4c and 4d). Furthermore, the average cell migration speed for MDA-MB-231 cells was found to be higher than that of the MCF-10A cell line (data not shown).

### Enhanced Experimental Control *via* Surface Functionalization and Pharmacological Delivery

During cell migration, parameters such as ECM composition, cellular integrin expression, integrin and substrate binding affinity can all directly influence the speed of cell movement [35]. Furthermore, cells encounter various ECM types *in vivo* [36]. To demonstrate the versatility of our device for simulating various environmental conditions, we showed the relationship between cell migration speed and ECM substrate by adsorbing various soluble ECM substrates to the interior channel surfaces. HOS cells migrated efficiently on collagen I/IV-, laminin-, and fibronectin- coated surfaces but to a lesser extent on hyaluronic acid-coated substrate (Figure S3b).

Paclitaxel and Latrunculin A (LA) are small-molecule inhibitors of tubulin [37] and actin [38] polymerization, respectively. Both microtubules and microfilaments are crucial for efficient cell movement as shown in traditional migration assays [37], [39]. To demonstrate the feasibility of assessing migratory potential after pharmacological inhibition using ECM-coated microchannels, we conditioned the assay medium with paclitaxel or LA at different dosages and analyzed HOS cell migration velocity inside 6 µm-wide microchannels (Figure S3c). Our results show that paclitaxel effectively suppressed cell migration speed by 30% and 70% at 0.2 µM and 2 µM, respectively. LA triggered a 50% reduction in cell migration speed at 0.2 µM, and completely inhibited cell movement at 2 µM. Our assay, therefore, is amenable to a range of technical applications in the study of individual cell migration and pharmacological testing, while providing precise real-time control of multiple experimental parameters.

### Fixed and Live-Cell Microscopy of Cellular Components

**Figure 5:**
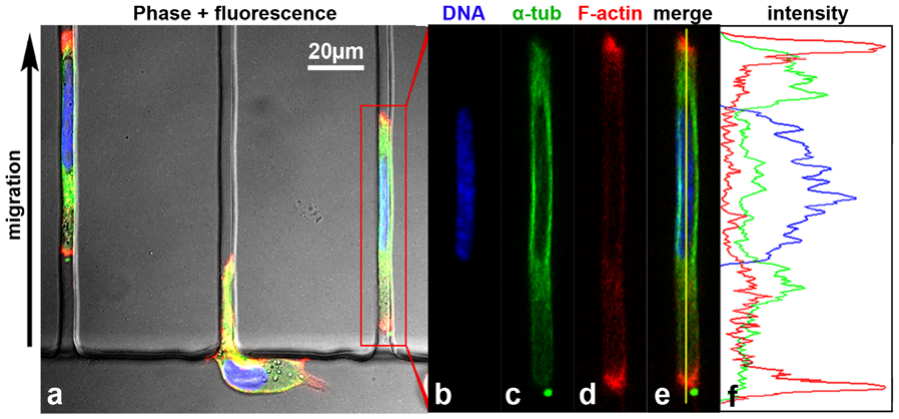
Confocal analysis of HOS cells migrating in 3 µm-wide microchannels. (a) A composite image (three-color fluorescence+differential interference contrast) depicts two cells inside 3 µm-wide microchannels, and a third cell entering a microchannel. (b) DAPI staining indicates an elongated nucleus distinct from the typical nuclear morphology on a flat 2D surface. (c) FITC-conjugated anti-α-tubulin labeling indicates that tubulin concentrates in a cytoplasmic and perinuclear region. (d) Phalloidin staining for F-actin revealed a specific enrichment of F-actin at the cell poles. (e) A fluorescence overlay image indicates the distinct subcellular localization of tubulin and F-actin, which is quantitatively represented in a color-coded intensity profile (sampled region is indicated by vertical yellow line) (f).

Because the PDMS migration chamber can be bonded to thin coverglass in place of standard microscope slides, both live and fixed cells can be visualized with high-resolution for immunofluorescence microscopy (Figure 5). HOS cells labeled with 4′, 6-diamidino-2-phenylindole (DAPI) underwent extensive nuclear deformation inside 3 µm-wide microchannels (Figure 5b). Co-labeling for α-tubulin and filamentous actin (F-actin) revealed a cytoskeletal reorganization of cells inside restrictive microchannels. Cytoplasmic microtubules were found to concentrate in a peri-nuclear region (Figure 5c), while F-actin was enriched at cell poles and along the lateral channel corners (Figure 5d and Figure S4). Using a microscope stage-top live cell incubator setup, fluorescent live-cell imaging is also feasible, as demonstrated by the visualization of GFP-actin dynamics [40] and organelle polarization [41] (RFP-Golgi) of migrating HOS cells in real-time (Movie S3).

### Internal Comparison of Different Cell Types to Determine Inherent Migratory Potential

**Figure 6:**
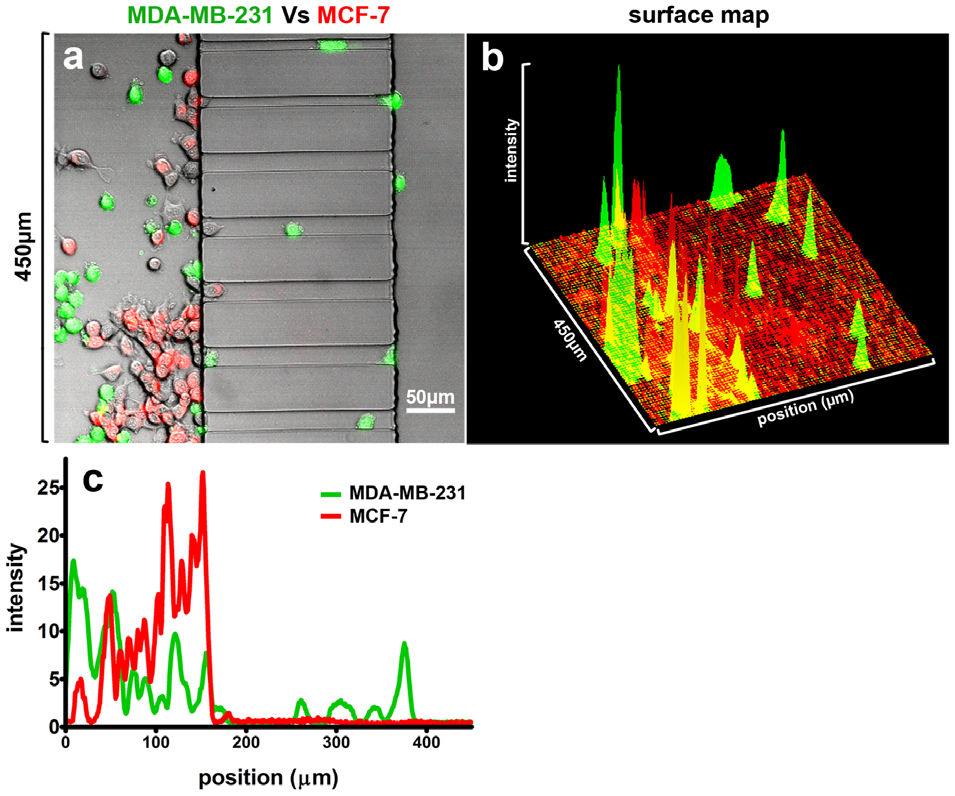
Direct comparison of multiple cell types in an enclosed migration system. (a) A representative image of mixture of MCF-7 and MDA-MB-2321 cells migrating through 20 µm and 10 µm wide microchannels. A surface map of fluorescence intensity (b) and horizontal intensity profile (c) are shown as adiditonal methods for quantifying the extent of migration for each cell type (horizontal axis of panel (c) drawn to scale to correspond with cell position in panel (a)).

We tested the feasibility of using our migration chamber to distinguish the locomotive potential of different cell types by directly comparing the ability of non-metastatic MCF-7 and invasive MDA-MB-231 breast tumor cell lines to migrate through microchannels. Cells were pre-labeled with two spectrally distinct fluorescent markers: a red fluorescent Golgi marker (MCF-7) and the green vital dye, CMFDA (MDA-MB-231); cells were then mixed in a 1∶1 ratio and introduced into a single microfluidic migration chamber. After 3 h of migration, the cells were fixed and analyzed by confocal microscopy. We observed that, for all channel widths, MDA-MB-231 cells migrated significantly further than MCF-7 cells (Figure 6). We repeated the experiment with reversed fluorescent labels, and found that metastatic MDA-MB-231 cells still migrated faster and farther t`han the non-metastatic MCF-7 cells independently of the labeling methods used (data not shown). These observations demonstrate that our microchannel device can be useful for comparing two or more cell types in a single enclosed system. However, we should be cautious when interpreting the results from experiments involving the use of heterogeneous cell populations, as different cell types may mutually influence one another *via* paracrine signaling. Heneweer et al. demonstrated that co-culture of fibroblasts with MCF-7 cells resulted in a paracrine feedback loop in an *in vitro* breast cancer model [42]. By the same token, co-culture of heterogeneous cell types in a single migration device could provide a more realistic environment in which to study how tumor cells and interstitial tissue cells interact during migration. Intermixing of cancer cells with immune cells, such as tumor-associated macrophages (TAMs), has been shown to facilitate metastasis, but the functional relationship underlying this correlation is unknown [43]. The device presented here could be useful in examining this mechanism in a controlled setting.

## Discussion

Herein, we demonstrate the use of a PDMS-based microfluidic migration chamber for the real-time observation of chemotaxis of cell populations at single cell resolution. Our design offers major improvements over conventional migration assays, and addresses some of the key deficiencies of current sophisticated methodologies used in the study of cell locomotion. First, our device provides a dynamic optical range of study, which permits users to track the movement of large cell populations at single cell resolution. This is accomplished by a microscope automation technique that enables the sequential capture of multiple fields of view arranged sequentially across the length of the microchannel array. This process can be repeated for up to four cell migration chambers, each of which can accommodate the simultaneous chemotaxis of several hundred cells. The resulting data can then be analyzed individually, or seamlessly integrated into a montage data file (Movie S1). Because of the excellent optical qualities of the 1 mm coverglass that forms the chamber floor, cells can be easily fixed and analyzed by indirect immunofluorescence at the conclusion of a time-lapse experiment. Additionally, this migration chamber allows the researcher to fine-tune the physical properties of the migratory environment, and can thus be used to study cellular responses to physical cues in a highly controlled and highly repeatable fashion. Because the dimensions and overall shape of the microchannels can be easily adjusted through the use of custom photomasks, the user can choose from a range of geometric configurations to study the movement of cells across planar 2D surfaces as well as through confined spaces that simulate restrictive 3D physiological environments [44]. Microchannels may also be useful in establishing barriers to lateral cell movement to promote unidirectional migration, which streamlines the analysis of cell velocity, net displacement, and other quantitative parameters of cell migration. Furthermore, channel surfaces can be easily functionalized through adsorption of different migratory substrates or filled with reconstituted ECM matrices to study proteolysis, offering an additional measure of experimental control.

While microfluidic systems to the study of cell locomotion have been reported [27], [28], they lack a chemotactic source and thus fail to induce directed cell migration. In the absence of a chemotactic stimulus, cells exhibited spontaneous motion and, upon reaching the distal end of the microchannels, turned back and continued migrating towards the center of the channels [27], [28]. We therefore sought to provide the means to study directional cellular locomotion in a physiological context by incorporating a chemotactic gradient. The method presented herein relies on the inherent properties of non-mixing laminar flow of microfluidic streams, and therefore does not require cumbersome and expensive external pump systems to induce persistent chemotaxis. The migration chamber described here also enables the direct comparison of two or more cell types (e.g., knockdown vs. parental cell types, or pharmacologically-treated vs. untreated cells) within an enclosed system, which helps to minimize experimental variability. Finally, the device is both simple and inexpensive to fabricate, and requires very small volumes of media (≤100 µL per inlet) even for long-duration experiments. Though we minimize evaporation from open inlets and outlets through control of relative humidity of the stage-top incubator, further evaporation may occur during such long-duration experiments. To control this, our device can easily be sealed from the top with a thin layer of PDMS. This sealing layer would prevent free evaporation of media sources while still allowing free gas exchange. Therefore, our novel design offers major improvements over current methods for the study of cell migration by combining the optical clarity and technical ease of *in vitro* assays with the enhanced physiological relevance of *in vivo* models. This migration chamber therefore integrates the strengths of many different techniques into one system, and could be a useful tool in a wide range of biological and biomedical research applications.

Using a series of PDMS microchannels of varying dimensions, we examined the influence of geometric confinement on cell migration. Our results indicate that cells migrate with disparate migration speeds as a function of channel width. Similarly, cell morphology was markedly influenced by channel size. In larger microchannels (>10 µm), lamellipodia and filopodia were observed, which are typical “2D” earmarks of mesenchymal motility. However, in narrow microchannels as small as 3 µm-wide, those distinct “2D” characteristics were lost as cells assumed a distinctly non-mesenchymal morphology. While migrating in 3 µm-wide channels, the cell body established contact with all four channel walls. As a result, the narrow channels constitute an environment that closely approximates a 3D setting. The differences observed in the mode of cell migration (*i.e.*, mesenchymal vs non-mesenchymal in narrow channels) was further emphasized by a reorganization of F-actin and α-tubulin for cells inside 3 µm-wide microchannels. The mechanism by which this occurs as cells transition from unrestricted 2D to 3D microenvironments is presently under investigation in our laboratory.

## Materials and Methods

### Cell Culture and Reagents

All chemicals, unless otherwise noted, were purchased from Invitrogen. Human Osteosarcoma cells (HOS.pBABE-puro) were obtained from the NIH AIDS Research and Reference Reagent Program, Division of AIDS, NIAID, NIH. HOS cells were grown in Dulbecco's Modified Eagle Medium (DMEM) supplemented with 10% FBS (Sigma) and 1% Penicillin-Streptomycin. The MCF-7 cell line was obtained from ATCC (American Type Culture Collection). MDA-MB-231 and MCF-10A cell lines were obtained from the Physical Science in Oncology Center of the National Cancer Institute. MCF-7 and MDA-MB-231 cells were cultured in DMEM supplemented with 10% FBS (Atlanta Biologicals) and 1% Penicillin-Streptomycin. MCF-10A cells were cultured in DMEM supplemented with 5% horse serum (Atlanta Biologicals), hydrocortisone (0.5 µg/ml, Sigma), hEGF (20 ng/ml), insulin (10 µg/ml, Sigma), cholera toxin (100 ng/ml, Sigma), and 1% Penicillin-Streptomycin.

### Fabrication of the Microfluidic Migration Chamber

The cell migration chamber was fabricated by standard photolithography. A chrome coated soda-lime glass transparency mask (Advance Reproductions Corp.) was designed with an array of rectangular dark features of fixed width (50 µm), with variable transparent separation distances, ranging from 3 µm to 50 µm (mask #1). SU-8 2010 photoresist (Microchem) was spin-coated at 3500 rpm onto a silicon wafer to achieve a thickness of 10 µm. It was then exposed to UV light through mask #1, baked on hot plate at 95°C, and processed with developer to generate a first layer of photoresist, which eventually formed the microchannels. The silicon wafer with crosslinked first layer of photoresist was primed with SU-8 2002 prior to spin-coat with a thicker second layer of SU-8 2025 (50 µm in height) at 1750 rpm. A high-resolution transparency film (5080 dpi) of features containing parallel main channels for cell seeding and chemokine gradient generation was designed (Adobe Photoshop) and printed (Pageworks, mask #2). Mask #2 was aligned perpendicularly to the first feature and exposed to UV light, baked, and processed with developer to generate 2^nd^ layer of photoresist, which represents the precursor for chemokine and cell seeding channels. Poly(dimethyl siloxane) (PDMS, Dow Chemical) replicas were obtained by casting mixture of PDMS prepolymer and curing agents (10∶1) over the photoresist wafer mold. A 3 mm hole puncher (Ted Pella, Inc.) was used to create flow ports. The PDMS replica was then irreversibly sealed to a rectangular glass slide or coverglass (Electron Microscopy Sciences) of standard length and width (25 mm×75 mm) via brief treatment with oxygen plasma (20 s) in a plasma cleaner (Harrick Plasma) to form the cell migration chamber. Scanning electron microscopy was used to characterize the dimensions of the microchannels. Images were captured with a JEOL JSM-6700F cold cathode field emission SEM in LEI/SEI mode.

### Cell Loading and Migration Experiment

After sealing the PDMS replica onto a clean glass slide, ECM (type I collagen (BD Bioscience), fibronectin (Sigma), laminin (Sigma), type IV collagen (Sigma) or hyaluronic acid (Sigma)) prepared at 20 µg/ml in phosphate buffered saline (PBS), were introduced into the migration chamber by capillary effect, and incubated thereafter for 1 h at 37°C. Microchannels were washed with PBS prior to addition of 50 µL of cell suspension (5×10^6^ cells/ml). Cells were then incubated for 5 min at 37°C to allow initial cell seeding. The cell suspension from the cell inlet port was then removed and replaced by serum free media (100 µL). The topmost inlet port of the chemokine gradient generator was filled with 100 µL of media containing prescribed concentrations of FBS while the other 2 inlet ports were filled with 100 µL of serum free media. In select experiments, latrunculin A (0.2 µM or 2 µM, sigma) or paclitaxel, commercially known as Taxol (0.2 µM or 2 µM), was added into the assay media. The migration chamber was then moved to a stage-top live cell incubator (Okolab, Italy) with a controlled cell culture environment (CO_2_/air mixture, temperature, and relative humidity), mounted on a motorized stage of an inverted Eclipse Ti microscope (Nikon). Migration experiments were visualized with a DS-Fi1 camera head and a 10× objective. NIS-Elements was set to capture images every 10 min for the duration of each live cell experiment. Cell migration speed was analyzed using the NIS-Elements add-on Tracking module.

### Immunofluorescence Microscopy

At the conclusion of a migration experiment, cells were fixed inside the microfluidic migration chamber by removing the cell medium and adding a solution of 3.7% formaldehyde (J. T. Baker) in PBS. Cells were fixed for 30 min, and then washed with PBS three times. Triton X-100 (0.1%, Sigma) was used to permeabilize cell membranes, and blocking was achieved using 1% BSA in PBS for 30 minutes. A mixture of 4′,6-diamidino-2-phenylindole (DAPI, 1 µg/ml, Roche), Alexa Fluor 488 conjugated anti-a-tublulin (5 µg/ml, eBioscience), and Alexa Fluor 568 conjugated phalloidin (1∶500 dilution, Invitrogen) in 1% BSA was then introduced into the migration chamber and incubated for 1 hr. After three PBS rinses, the migration chamber ports were filled with antifade reagent. Confocal microscopy was performed with a Zeiss LSM 510 META Confocal with 63× oil-immersion and 10× dry objectives. Images were captured using Zeiss LSM software, and analyzed in ImageJ (Bethesda, MD).

### Co-migration Experiments with MCF-7 and MDA-MB-231

MCF-7 cells were transiently transfected with CellLight Golgi-RFP BacMam, according to the manufacturer's instructions (Invitrogen, CA) 24 h before the onset of the migration experiment. MCF-7 cells were detached by 0.05% trypsin, washed in PBS and suspended in serum-free medium. MDA-MB-231 cells were incubated with the green vital dye, 5-chloromethylfluorescein diacetate (CMFDA, 2 µM) for 30 min at culture conditions, trypsinized, washed and re-suspended in serum-free medium. Pre-labeled MCF-7 and MDA-MB-231 cells were mixed at a 1∶1 ratio and added to the inlet port of a migration chamber, which was transferred to cell culture incubator to allow for initial cell seeding. The cell suspension was removed after 3 min and the inlet ports were replenished with fresh media. The migration chamber was then placed back in cell incubator for 3 h, at which point cells were fixed by adding 3.7% formaldehyde to the chamber inlets prior to imaging via confocal microscopy. For control experiments, MDA-MB-231 cells were transfected with CellLight reagent while MCF-7 cells were labeled with CMFDA to ensure the staining processes did not influence migratory ability.

### Image and Data Analysis

Confocal images were captured with Zeiss LSM 510 META confocal scanner, and Image J (NIH, Bethesda, MD) was used for subsequent image processing and analysis. Cell migration speed was calculated from cell displacement of cell centroid relative to the elapsed time using a particle-tracking algorithm in NIS-Elements: Tracking (Nikon, Japan). Statistical significance was determined using unpaired Student's t-test from GraphPad software (La Jolla, CA); *p* values<0.05 were interpreted as significant. Where shown, error bars depict standard error of the mean.

## Supporting Information

Figure S1 **Microfabrication of the cell migration chamber.** (a) A silicon wafer is spin-coated with SU-8 photoresist, selectively exposed to UV light through photomask #1, and subsequently processed with SU-8 developer to remove uncrosslinked SU-8 and raise microchannel-negative features (b). The silicon wafer is then spin-coated with a second thicker layer of SU-8. Photomask #2 is aligned so that the large ‘horizontal’ channels are perpendicular to the ‘vertical’ first feature, exposed to UV light (c), and process with SU-8 developer to generate the 2nd layer of photoresist (d). PDMS prepolymer is mixed with curing agent and poured onto the mold to generate a negative replica of the photoresist features (e). After polymerization, the PDMS layer is peeled off from the mold, hole-punched to generate inlet and oulet ports, and irreversibly sealed to a glass slide to form a complete cell migration chamber (f). ECM solution is then subsequently added to saturate the interior of the device.
(TIF)

Figure S2 **Scanning electron microscopic characterization of photoresist features on silicon wafer.** SEM was used to measure the width of the negative microchannel features (W_M_) as compared with the desired feature width (W_D_) of 10 (a), 6 (b), and 3 µm (c).
(TIF)

Figure S3 **Cell migration speed is independent of channel length but dependent on ECM substrate and drug treatments.** (a) HOS cell migration speed in 6 µm-wide microchannels did not vary with different channel lengths (L = 100, 200, and 400 µm). (b) HOS cells migrated with variable efficiency on different types of ECM coated 6 µm-wide microchannels. The cell migration speeds were compared relative to type I collagen coated microchannels. (c) Both latrunculin A (0.2 µM or 2 µM) and paclitaxel (Taxol, 0.2 µM or 2 µM) are shown to be effective chemical treatments to inhibit cell migration as relative to untreated cells.
(TIF)

Figure S4 **Confocal analysis and volumetric rendering of HOS cell inside a microchannel.** An HOS cell migrating through 3 µm-wide microchannel was labeled with a fluorescent phalloidin conjugate and analyzed by confocal microscopy. Volumetric rendering indicates a preferential localization of F-actin at the cell front and trailing edges, and at channel corners along the long axis of the migrating cell.
(TIF)

Movie S1 **A ‘panoramic’ view of an entire HOS cell population migrating in a single microchannel device.** This movie is a montage of 10 contiguous fields of view imaged over the course of 8 h.
(AVI)

Movie S2 **HOS cells migrate into and exit 6 µm and 3 µm-wide microchannels.**
(AVI)

Movie S3 **Live-cell fluorescence tracking of HOS cells transfected with GFP-actin or RFP-golgi.**
(AVI)
