# Supplementary figures and images for: Chemotaxis of Cell Populations through Confined Spaces at Single-Cell Resolution
Source: PLoS One. 2012 Jan 18;7(1):e29211. doi: 10.1371/journal.pone.0029211 (PMC3261140; doi:10.1371/journal.pone.0029211)

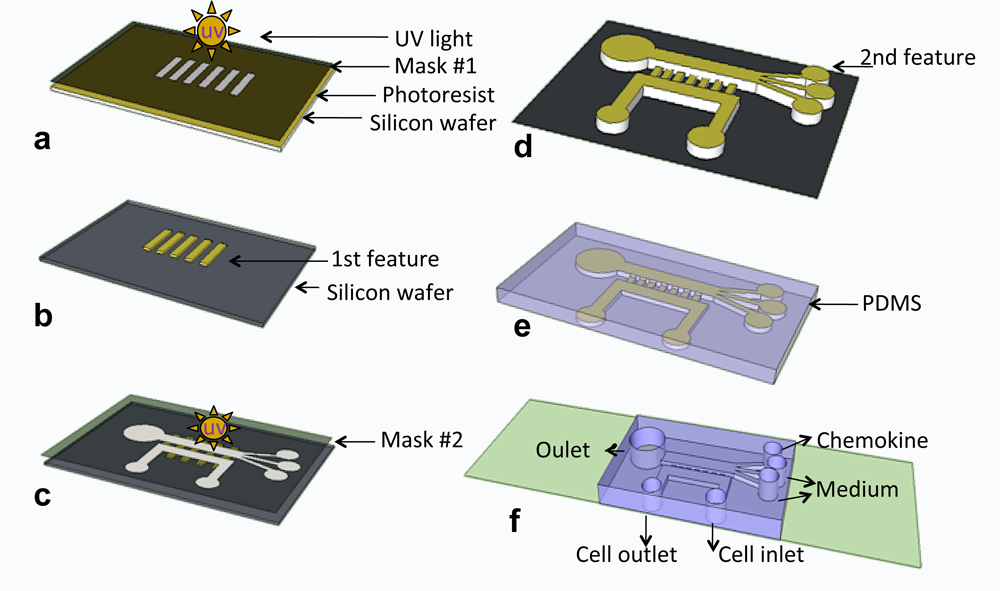

Supplement: Figure S1 — Microfabrication of the cell migration chamber. (a) A silicon wafer is spin-coated with SU-8 photoresist, selectively exposed to UV light through photomask #1, and subsequently processed with SU-8 developer to remove uncrosslinked SU-8 and raise microchannel-negative features (b). The silicon wafer is then spin-coated with a second thicker layer of SU-8. Photomask #2 is aligned so that the large ‘horizontal’ channels are perpendicular to the ‘vertical’ first feature, exposed to UV light (c), and process with SU-8 developer to generate the 2nd layer of photoresist (d). PDMS prepolymer is mixed with curing agent and poured onto the mold to generate a negative replica of the photoresist features (e). After polymerization, the PDMS layer is peeled off from the mold, hole-punched to generate inlet and oulet ports, and irreversibly sealed to a glass slide to form a complete cell migration chamber (f). ECM solution is then subsequently added to saturate the interior of the device. (TIF) [file pone.0029211.s001.tif]

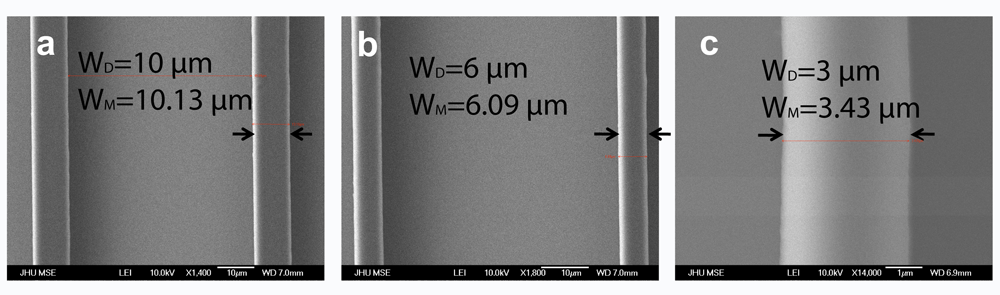

Supplement: Figure S2 — Scanning electron microscopic characterization of photoresist features on silicon wafer. SEM was used to measure the width of the negative microchannel features (WM) as compared with the desired feature width (WD) of 10 (a), 6 (b), and 3 µm (c). (TIF) [file pone.0029211.s002.tif]

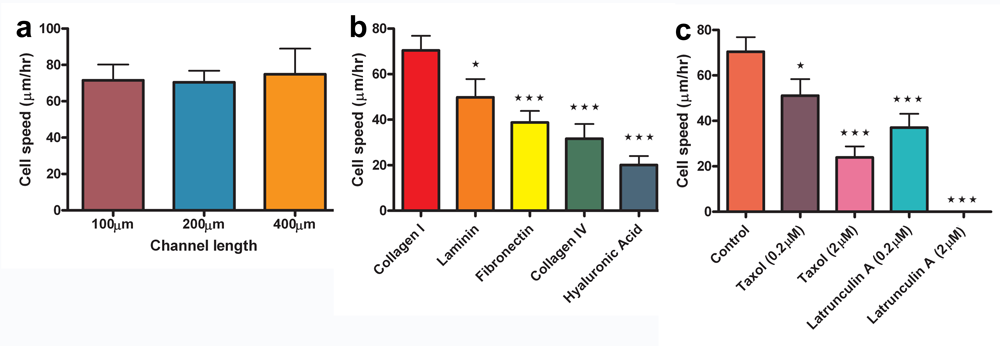

Supplement: Figure S3 — Cell migration speed is independent of channel length but dependent on ECM substrate and drug treatments. (a) HOS cell migration speed in 6 µm-wide microchannels did not vary with different channel lengths (L = 100, 200, and 400 µm). (b) HOS cells migrated with variable efficiency on different types of ECM coated 6 µm-wide microchannels. The cell migration speeds were compared relative to type I collagen coated microchannels. (c) Both latrunculin A (0.2 µM or 2 µM) and paclitaxel (Taxol, 0.2 µM or 2 µM) are shown to be effective chemical treatments to inhibit cell migration as relative to untreated cells. (TIF) [file pone.0029211.s003.tif]

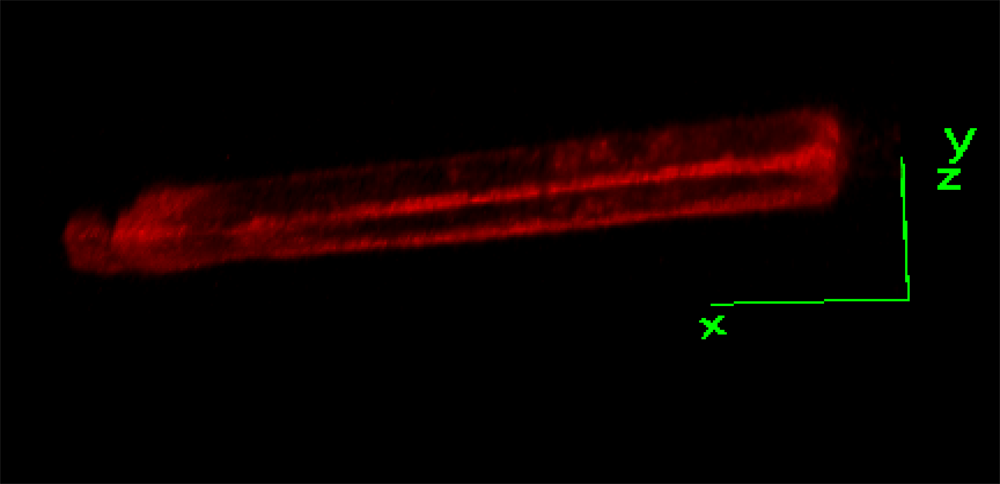

Supplement: Figure S4 — Confocal analysis and volumetric rendering of HOS cell inside a microchannel. An HOS cell migrating through 3 µm-wide microchannel was labeled with a fluorescent phalloidin conjugate and analyzed by confocal microscopy. Volumetric rendering indicates a preferential localization of F-actin at the cell front and trailing edges, and at channel corners along the long axis of the migrating cell. (TIF) [file pone.0029211.s004.tif]
